# Knockdown of circ-FANCA alleviates LPS-induced HK2 cell injury via targeting miR-93-5p/OXSR1 axis in septic acute kidney injury

**DOI:** 10.1186/s13098-021-00625-8

**Published:** 2021-01-19

**Authors:** Heyun Li, Xia Zhang, Peng Wang, Xiaoyan Zhou, Haiying Liang, Caoni Li

**Affiliations:** 1 Department of Critical Care Medicine, No. 215 Hospital of Shaanxi Nucler Industry, Xianyang, 712000 China; 2 Department of Rulmonary and Critical Care Medicine, Hanzhong City Central Hospital of Shaanxi Province, Hanzhong, 723000 China; 3 Department of Respiratory Medicine, Baoji Central Hospital, Baoji, 721008 China; 4 Department of Vascular Intervention, Shaanxi Hospital of Traditional Chinese Medicine, Xi’an, 710003 China; 5 Department of Rulmonary and Critical Care Medicine, Shaanxi Hospital of Traditional Chinese Medicine, Xi’an, 710003 China; 6 Department of Hematology, Shangluo Central Hospital, No. 148 Beixin Street, Shangluo, 726000 China

**Keywords:** Sepsis, AKI, circ-FANCA, miR-93-5p, OXSR1

## Abstract

**Background:**

Sepsis is life-threatening disease with systemic inflammation and can lead to various diseases, including septic acute kidney injury (AKI). Recently, diverse circular RNAs (circRNAs) are considered to be involved in the development of this disease. In this study, we aimed to elucidate the role of circ-FANCA and the potential action mechanism in sepsis-induced AKI.

**Methods:**

HK2 cells were treated with lipopolysaccharide (LPS) to establish septic AKI cell model. The expression of circ-FANCA, microRNA-93-5p (miR-93-5p) and oxidative stress responsive 1 (OXSR1) mRNA was determined by quantitative real-time polymerase chain reaction (qRT-PCR). Cell viability was assessed using cell counting kit-8 (CCK-8) assay. Cell apoptosis and cell cycle distribution were measured by flow cytometry. The inflammatory response was monitored according to the release of pro-inflammatory cytokines via enzyme-linked immunosorbent assay (ELISA). The activities of oxidative indicators were examined using the corresponding kits. Dual-luciferase reporter assay and RNA immunoprecipitation (RIP) assay were applied to validate the interaction between miR-93-5p and circ-FANCA or OXSR1. Protein analysis was conducted through western blot.

**Results:**

Circ-FANCA was upregulated in septic AKI serum specimens and LPS-treated HK2 cells. Functionally, circ-FANCA knockdown facilitated cell proliferation and restrained apoptosis, inflammation and oxidative stress in LPS-triggered HK2 cells. Further mechanism analysis revealed that miR-93-5p was a target of circ-FANCA and circ-FANCA modulated LPS-induced cell damage by targeting miR-93-5p. Meanwhile, miR-93-5p overexpression repressed LPS-treated HK2 cell injury by sponging OXSR1. Furthermore, circ-FANCA regulated OXSR1 expression by sponging miR-93-5p. Besides, exosome-derived circ-FANCA was upregulated in LPS-induced HK2 cells, which was downregulated by GW4869.

**Conclusion:**

Circ-FANCA knockdown attenuated LPS-induced HK2 cell injury by regulating OXSR1 expression via targeting miR-93-5p.

## Background

Sepsis is a complicated systemic disease with high morbidity and fatality rate, which is caused by infection and can evoke organ dysfunction [1, 2]. Acute kidney injury (AKI) is a frequent sepsis-related complication with high incidence accompanied by acute inflammation and tissue injury [3]. Previous study has suggested that natural killer T (NKT) cells were involved in the infectious and autoimmune diseases [4]. Meanwhile, CD4+ T cells and CD4+ T cells secreting interferon-gamma (IFN-γ) positive lymphocytes could participate in immune response of HIV infection and mycoplasmal infection [5–7]. Similarly, the accumulation of multifarious immune cells including NKT cells, CD4+ T cells, B cells and IFN-γ-producing neutrophils are closely associated with the pathogenesis of AKI [8–10]. Lipopolysaccharide (LPS)-induced sepsis is a primary cause of AKI in critically ill patients [11, 12]. Therefore, LPS is always utilized to construct septic AKI cell models in renal cells in vitro [13]. For the intricate pathogenesis of sepsis-induced AKI, exploring the latent mechanisms and effective therapeutic strategies are of great significance for AKI treatment.

Circular RNAs (circRNAs) are a widespread class of non-coding RNAs with closed-loop structures and high stabilization [14]. Accumulating evidence stated that numerous circRNAs are closely associated with multiple human diseases [15], including sepsis [16]. For example, circVMA21 played a crucial part in sepsis-associated AKI via microRNA-9-39 (miR-9-39) [17]. Circ-FANCA (hsa_circ_0040994) is produced from precursor mRNA FANCA, and it is identified to be highly expressed in sepsis-stimulated AKI [18]. However, the function of circ-FANCA in the progression of sepsis-induced AKI and the underlying mechanisms involved remain unknown.

MicroRNAs (miRNAs) are a class of small RNAs without the ability to code proteins, and can modulate gene expression at the post-transcriptional levels [19]. MiRNAs have been unveiled to be involved in sepsis-induced AKI [20]. For instance, Qin et al. reported that miR-133a was able to relieve sepsis-induced renal damage through binding to BNIP3L [21]. Meanwhile, He et al. declared that miR-93-5p was downregulated in LPS-treated HK2 cells and its overexpression weakened sepsis-induced AKI [22]. Nonetheless, the potential target relationship between circ-FANCA and miR-93-5p in sepsis-stimulated AKI has been unexplored.

In the current study, we constructed septic AKI model in HK2 cells with the stimulation of LPS. The expression pattern of circ-FANCA and its function were investigated in LPS-treated HK2 cells. Meanwhile, circ-FANCA was found to act a sponge for miR-93-5p. Mechanistically, the regulatory function of circ-FANCA/miR-93-5p/mRNA network was explored, which might provide a new perspective for the pathogenesis of sepsis-related AKI.

## Materials and methods

### Serum samples

The serum specimens were collected from 19 fasting septic AKI patients and 19 fasting healthy subjects at No. 215 Hospital of Shaanxi Nucler Industry. These serum specimens were acquired by centrifugation and then preserved at − 80 °C prior to use. Written informed consent from all participants. The clinicopathologic characteristics of sepsis-AKI patients was listed in Table 1.

**Table 1:** Clinicopathologic features of Sepsis-AKI patients

Parameters	Normal group	Sepsis-AKI group
Number of patients	19	19
Gender (male/female)	10/9	11/8
Age (years)	52.3 ± 6.3	58.6 ± 8.6
Serum creatinine (µmol/L)	65.3 ± 5.6	312.5 ± 29.6
Blood urea nitrogen (mmol/L)	4.3 ± 0.6	23.6 ± 1.7
CRP (mg/L)		185.6 ± 21.3
Calcitonin original (ng/L)		22.3 ± 3.6

### Cell culture and LPS induction

Human renal tubule epithelial cells (Human kidney-2, HK2) were purchased from Procell (Wuhan, China) and cultured in Minimum Essential Medium (MEM; Procell) plus 10% fetal bovine serum (FBS; Procell) and 1% penicillin–streptomycin (Procell) in a 37 °C, 5% CO_2_ humid incubator.

HK2 cells were treated with LPS at different concentrations (0, 2.5, 5 and 10 µg/mL) for 24 h. For the establishment of sepsis-related AKI model in vitro, HK2 cells in the following experiments were exposed to 5 µg/mL LPS (Solarbio, Beijing, China) for 12 h, while the control HK2 cells were cultivated with MEM medium simultaneously.

### Cell transfection

Small interfering RNA (siRNA) targeting circ-FANCA (si-circ-FANCA) was synthesized to mediate circ-FANCA knockdown, with si-NC served as negative control. The overexpression vector of circ-FANCA was constructed through cloning the full length sequence of circ-FANCA into the empty pCD5-ciR (Geneseed Biotech, Guangzhou, China), with a non-target pCD5-ciR vector as a negative control. The mimic and inhibitor of miR-93-5p (miR-93-5p and anti-miR-93-5p) were respectively constructed for miR-93-5p overexpression and knockdown, with miR-NC and anti-miR-NC acted as corresponding negative controls. Oxidative stress responsive 1 (OXSR1) was amplified and cloned into pcDNA3.1 vector (pcDNA; Invitrogen, Carlsbad, CA, USA) to construct OXSR1 overexpression plasmid (OXSR1), with pcDNA empty vector as the control. LPS-treated HK2 cells were planted into 96-well plates overnight to 70% confluence and then transduced with above oligonucleotides and plasmids obtained from RiboBio (Guangzhou, China) for 48 h. Cell transfection was executed using Lipofectamine 3000 Reagent (Invitrogen).

### The quantitative real-time polymerase chain reaction (qRT-PCR)

Total RNA was acquired from serums and cells utilizing via Trizol reagent (Invitrogen), and reversely transcribed into cDNA using Primer Script RT reagent kit (Takara, Dalian, China) or miScript Reverse Transcription Kit (Qiagen, Hilden, Germany). QRT-PCR assay was manipulated using cDNA, SYBR Premix Ex Taq II (Takara) and specific primers (Sangon, Shanghai, China) on PCR system. Relative expression was evaluated via the 2^−∆∆Ct^ method with glyceraldehyde-phosphate dehydrogenase (GAPDH) and U6 as internal references. The sequences of primers were listed as below: circ-FANCA, 5′-CAGTTTGCCAGCGATTTCCT-3′ (forward, F) and 5′-TAGCTCCTCTCTCTCGCAGT-3′ (reverse, R); FANCA, 5′-CAGAACCCAACTCTGCTGAGGA-3′ (F) and 5′-ATCACTGCCACCTGTGCCGATA-3′ (R); miR-93-5p, 5′-AGCAGTCAGTAGTTGGTCCTTTG-3′ (F) and 5′-CCATCAGTCCCGTCTTGAAAC-3′ (R); OXSR1, 5′-AAAGACGTTTGTTGGCACCC-3′ (F) and 5′-GCCCCTGTGGCTAGTTCAAT-3′ (R); GAPDH, 5′-TGCACCACCAACTGCTTAGC-3′ (F) and 5′-GGCATGGACTGTGGTCATGAG-3′ (R); U6, 5′-CTCGCTTCGGCAGCACA-3′ (F) and 5′-AACGCTTCACGAATTTGCGT-3′ (R).

### Subcellular fractionation location

The nuclear or cytoplasmic RNA fraction was isolated from HK2 cells by mirVana PARIS Kit (Invitrogen). Then, the separated RNA from each of the fractions was used for qRT-PCR analysis to detect the expression of circ-FANCA, with GAPDH and U6 as the internal references for cytoplasmic and nuclear fractions, respectively.

### Ribonuclease (RNase) R digestion

To assess the stability of circ-FANCA, RNase R (Epicentre Technologies, Madison, WI, USA) was utilized to digest linear RNA. In brief, total RNA (2 μg) was incubated with or without RNase R (3 U/μg) for 0.5 h at 37 °C. After that, the treated RNA was used for qRT-PCR analysis to measure the expression of circ-FANCA and linear FANCA.

### Cell counting kit-8 (CCK-8) assay

HK2 cells with various treatments were seeded into 96-well plates (1 × 10^4^ cells/well) and incubated for 48 h. Subsequently, 10 μL CCK-8 reagent (5 mg/mL; Beyotime, Shanghai, China) was pipetted into each well, and cells were maintained at 37 °C for another 2 h. The absorbance of each well at 450 nm measured on a microplate reader (Thermo Fisher Scientific, Waltham, MA, USA) was utilized for determining cell viability.

### Flow cytometry assay

Flow cytometry analysis was executed for the detection of cell cycle distribution and cell apoptosis. Cell apoptosis was evaluated by Annexin V-fluorescein isothiocyanate (FITC)/propidium iodide (PI) apoptosis detection kit (BD Biosciences, San Jose, CA, USA). In brief, HK2 cells were collected and resuspended in Annexin binding buffer at 48 h post-transfection. Subsequently, cells were double-stained with 5 μL Annexin V-FITC and 5 μL PI in the dark for 15 min. The apoptotic rate of HK2 cells was identified by flow cytometer (BD Biosciences).

For the determination of cell cycle distribution, transfected HK2 cells were collected and resuspended in PBS, then fixed with 70% ethanol at 4 °C for 2 h and incubated with PI staining solution (BD Biosciences) containing RNase in the dark at 37 °C for 20 min. Cell cycle distribution at different phases (G0/G1, S and G2/M) was assessed using flow cytometer (BD Biosciences).

### Enzyme-linked immunosorbent assay (ELISA)

The release of inflammatory cytokines interleukin-1β (IL-1β) and tumor necrosis factor α (TNF-α) in culture medium was determined using the corresponding ELISA kits against TNF-α and IL-1β (Beyotime) according to the user’s manual. The colorimetric changes were determined at 450 nm.

### Oxidative stress assay

Oxidative stress was evaluated by the levels and activities of oxidative indicators. In brief, after LPS stimulation and relevant transfection, the levels of superoxide dismutase (SOD) and malondialdehyde (MDA) in the supernatants of HK2 cells were measured using the SOD assay kit (Beyotime) or MDA assay kit (Beyotime) according to the instructions, respectively.

### Bioinformatics analysis

The potential miRNAs or mRNAs respectively targeted by circ-FANCA or miR-93-5p were predicted by the bioinformatics tool starBase v2.0 (http://starbase.sysu.edu.cn/).

### Dual-luciferase reporter assay

The wild-type (WT) sequences of circ-FANCA or OXSR1 3′UTR containing miR-93-5p binding sites were inserted into pGL3 Luciferase Reporter Vectors (Promega, Madison, WI, USA) to generate corresponding luciferase reporter plasmids, named as WT-circ-FANCA and OXSR1 3′UTR-WT, respectively. Similarly, the mutant-type (MUT) sequences of circ-FANCA or OXSR1 3′UTR lacking miR-93-5p binding sites were cloned into pGL3 vector (Promega), constructing MUT-circ-FANCA and OXSR1 3′UTR-MUT, respectively. Next, HK2 cells were cotransfected with above fusion plasmid and miR-93-5p or miR-NC. At 48 h post-transfection, the luciferase activity in these transfected cells was measured utilizing the Dual-Luciferase Reporter Assay System (Promega).

### RNA immunoprecipitation (RIP) assay

Magna RIP RNA-Binding Protein Immunoprecipitation kit (Millipore, Billerica, MA, USA) was utilized to conduct RIP assay. HK2 cells were lysed in RIP lysis buffer from above kit. Cell lysate was incubated with Argonaute 2 (Ago2) antibody (Abcam, Cambridge, MA. USA) or Immunoglobulin G (IgG) antibody (Abcam) conjugated with magnetic beads (Sigma-Aldrich, St. Louis, MO, USA) at 4 °C overnight. Then, the isolated RNA enrichment of circ-FANCA, miR-93-5p and OXSR1 in Ago2 or IgG group was measured by qRT-PCR.

### Western blot

Proteins from serums or cells were isolated by RIPA buffer (Millipore) and quantified by a BCA protein assay kit (Tiangen, Beijing, China). Next, equal amounts of protein were separated via sodium dodecyl sulfonate-polyacrylamide gel (Solarbio) electrophoresis and then transferred onto polyvinylidene difluoride membranes (PVDF, Beyotime). After blocked with 5% defatted milk for 1 h at indoor temperature, the membranes were incubated with primary antibodies against OXSR1 (1:2000; ab97694; Abcam), CD9 (1:1000; ab263019; Abcam), CD63 (1:1000; ab134045; Abcam) and GAPDH (1:10,000; ab181602, Abcam) overnight at 4 °C. Then, the proteins on the membrane were probed with the secondary antibodies (1:20000; ab205718 or ab205719; Abcam) at 37 °C for 2 h. The protein blots were visualized using BeyoECL Star Kit (Beyotime).

### Exosome isolation and disposition

The isolation of exosomes derived from LPS-treated HK2 cells was conducted using differential centrifugation. Briefly, the supernatant of cell culture medium of LPS-treated HK2 cells was collected and first centrifuged at 3000×*g* for 20 min at the room temperature for removing cells or other debris. Then, the cell-free culture supernatant was treated with Total Exosome Isolation Reagent (Invitrogen) at 4 °C for 12 h and centrifuged at 100,000×*g* for 20 min at 4 °C for the removing of shedding vesicles and other vesicles with larger sizes. After collecting, the samples were centrifuged again at 4 °C for 70 min at 100,000×*g*, followed by gently removing the supernatant. Finally, the exosomal precipitate was obtained and re-suspended in PBS. Transmission electron microscopy (Hitachi, Tokyo, Japan) was used to monitor the shape of isolated exosomes.

HK2 cells were added with 40 μg/mL exosomes isolated from culture medium of LPS-stimulated HK2 cells or exosome inhibitor GW4869 (MedChemExpress, Shanghai, China). Cells treated with PBS served as control.

### Statistical analysis

The data were exhibited as mean ± standard deviation with at least three duplicates. Data statistical analysis was performed using GraphPad Prism 6.0 software (GraphPad Software, La Jolla, CA, USA). Difference analysis between 2 groups or among multiple groups was implemented through Student’s *t*-test or one-way analysis of variance followed by Tukey post hoc test. The linear correlations among circ-FANCA, miR-93-5p and OXSR1 were analyzed using Spearman’s correlation coefficient. It was defined as significant difference if *P *< 0.05.

## Result

### Circ-FANCA was highly expressed in septic AKI patients and LPS-stimulated HK2 cells

**Fig. 1:**
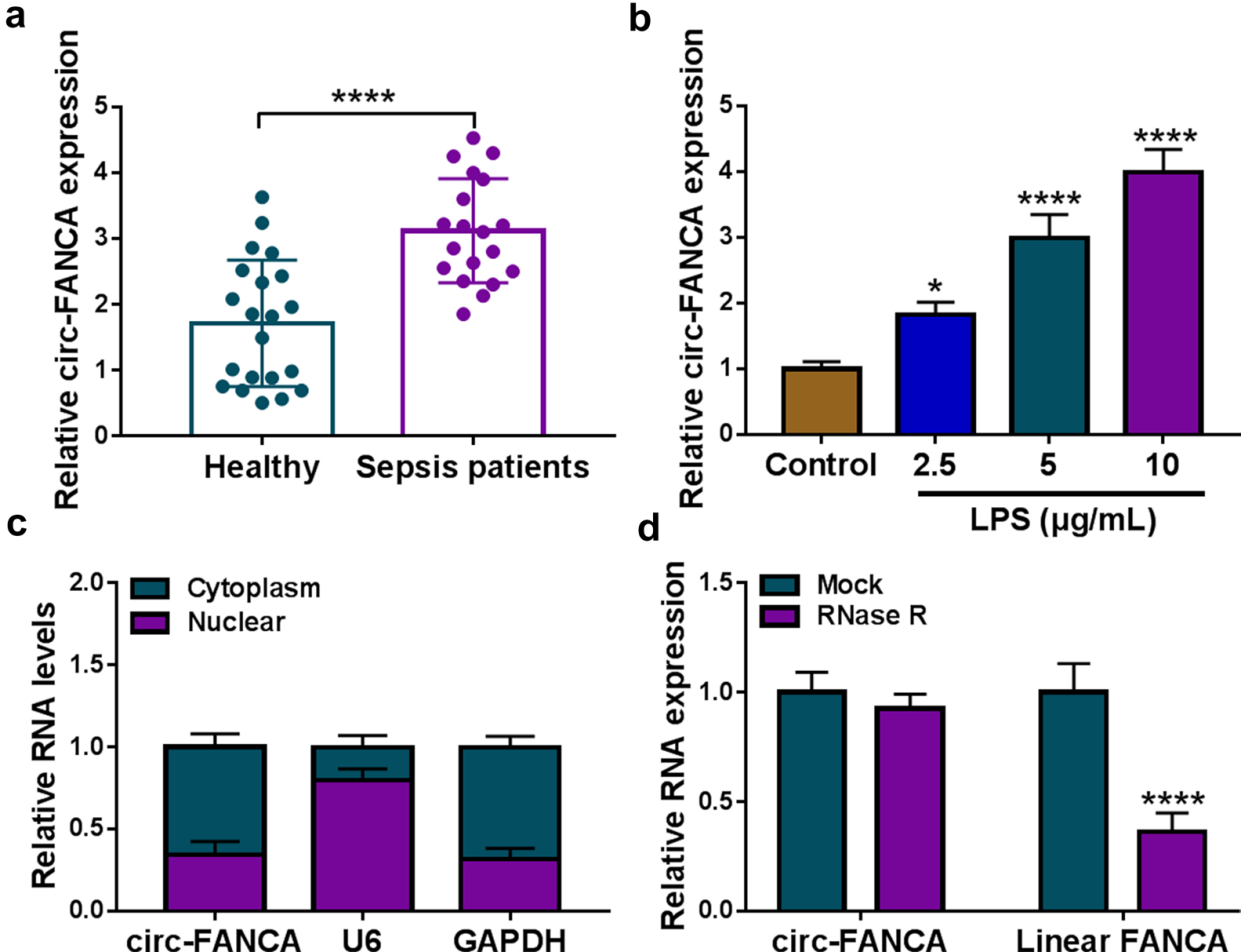
Circ-FANCA was overexpressed in septic AKI patients and LPS-treated HK2 cells. **a** The level of circ-FANCA was examined by qRT-PCR in serum samples of septic AKI patients (n = 19) and healthy controls (n = 19). **b** The expression of circ-FANCA in HK2 cells treated with different concentrations of LPS for 24 h was detected by qRT-PCR. **c** QRT-PCR assay was conducted for circ-FANCA expression in nuclear and cytoplasmic fractions. **d** After RNase R treatment, the levels of circ-FANCA and linear FANCA mRNA were determined using qRT-PCR. *P < 0.05, ****P < 0.0001

In the beginning, the expression level of circ-FANCA in the serum specimens obtained from septic AKI patients and healthy volunteers was determined by qRT-PCR analysis. The results showed that circ-FANCA was noticeably increased in the serum specimens from septic AKI patients in comparison with that in healthy controls (Fig. 1a). Next, the septic AKI model in vitro was established by treating HK2 cells with LPS. Firstly, the expression of circ-FANCA in HK2 cells after LPS exposure was observed. In LPS-treated HK2 cells, the expression of circ-FANCA was notably increased in a dose-dependent manner (Fig. 1b). 5 μg/mL LPS was selected for subsequent experiments. Then, the location and stability of circ-FANCA were detected using subcellular fractionation location and RNase R digestion assay. As shown in Fig. 1c, localization analysis indicated that circ-FANCA was mainly distributed in the cytoplasm of HK2 cells. Meanwhile, RNase R assay showed that the level of linear FANCA mRNA was significantly reduced by the digestion with RNase R, while circ-FANCA was resistant to RNase R, indicating circ-FANCA was a highly stable circular RNA (Fig. 1d). These observations indicated that circ-FANCA might be a functional molecule in septic AKI progression.

### Knockdown of circ-FANCA promoted proliferation and suppressed apoptosis and inflammation in LPS-treated HK2 cells

**Fig. 2:**
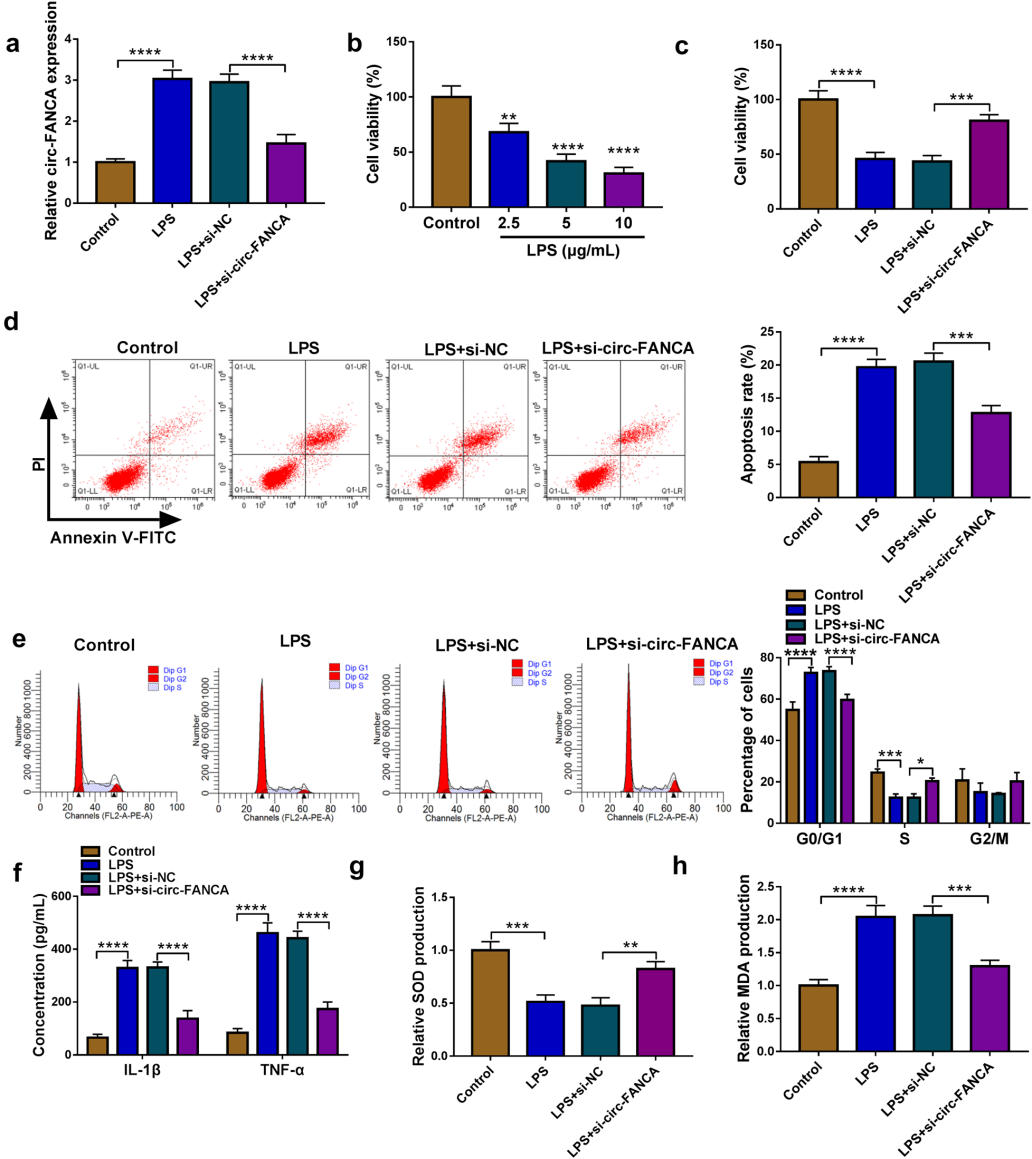
Circ-FANCA knockdown promoted proliferation and suppressed apoptosis and inflammation in LPS-treated HK2 cells. **a** The efficiency of siRNA-mediated circ-FANCA knockdown was examined by qRT-PCR in LPS-treated HK2 cells. **b** Cell viability in HK2 cells treated with different concentrations of LPS for 24 h was measured by CCK-8 assay. HK2 cells were divided into 4 groups: Control, LPS, LPS + si-NC and LPS + si-circ-FANCA. **c** Cell viability in LPS-treated HK2 cells with different transfection or not was measured by CCK-8 assay. **d**, **e** Cell apoptosis and cell period distribution were evaluated through flow cytometry in the above groups. **f** The release levels of inflammatory cytokines IL-1β and TNF-α were examined via ELISA using corresponding kits. **g**, **h** The levels of SOD and MDA in HK2 cells were measured by matched kits to assess oxidative stress. *P < 0.05, **P < 0.01, ***P < 0.001, ****P < 0.0001

To explore the role of circ-FANCA in LPS-treated HK2 cells, we knocked down its expression using si-circ-FANCA. Transient transfection of si-circ-FANCA distinctly decreased the expression of circ-FANCA in LPS-stimulated HK2 cells relative to si-NC group (Fig. 2a). Meanwhile, LPS treatment resulted in a prominent inhibitory effect on cell viability of HK2 cells in a dose-dependent manner (Fig. 2b). Next, HK2 cells were divided into 4 groups: Control, LPS, LPS + si-NC and LPS + si-circ-FANCA. CCK-8 assay presented that cell viability was restrained in LPS-induced HK2 cells, while si-circ-FANCA transfection recovered the viability (Fig. 2c). The flow cytometry assay suggested that cell apoptosis was promoted and cell cycle was arrested at G0/G1 stage, whereas circ-FANCA knockdown significantly suppressed these effects (Fig. 2d and e). Moreover, the release of inflammatory factors IL-1β and TNF-α induced by LPS was largely blocked by circ-FANCA silence using ELISA assay (Fig. 2f). Besides, the antioxidant capacity of HK2 cells was assessed by examining the levels of SOD and MDA via specific kits. As a result, it was found that LPS treatment reduced SOD level and elevated MDA level in HK2 cells, indicating that oxidative stress was enhanced in HK2 cells after LPS treatment; however, circ-FANCA knockdown could neutralize the effect of LPS on oxidative stress (Fig. 2g and h). Collectively, these data manifested that siRNA-mediated circ-FANCA silence mitigated LPS-induced HK2 cell injury, including proliferation inhibition, apoptosis promotion, inflammatory response and oxidative stress.

### Circ-FANCA directly interacted with miR-93-5p

**Fig. 3:**
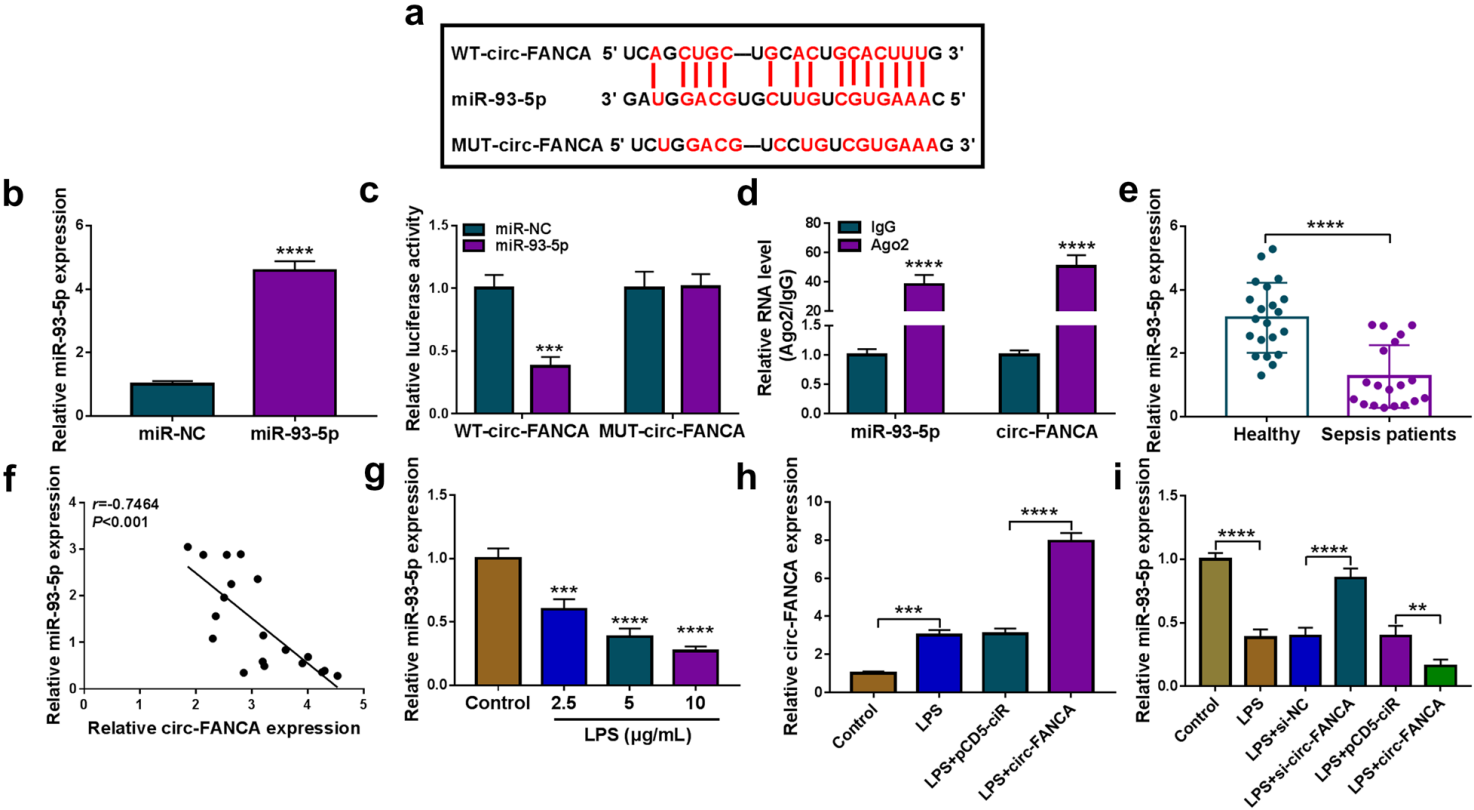
Circ-FANCA directly interacted with miR-93-5p. **a** The complementary sequence between circ-FANCA and miR-93-5p were exhibited. **b** The overexpression efficiency of miR-93-5p in HK2 cells was evaluated by qRT-PCR. **c**, **d** The targeted relationship between circ-FANCA and miR-93-5p was validated by dual-luciferase reporter assay and RIP assay. **e** The expression level of miR-93-5p in the serums of septic AKI patients (n = 19) and healthy controls (n = 19) was examined by qRT-PCR assay. **f** Spearman’s correlation coefficient analysis was performed to estimate the correlation between circ-FANCA and miR-93-5p in the serums of septic AKI patients. **g** The level of miR-93-5p in LPS-stimulated HK2 cells was determined by qRT-PCR assay. **h** The expression level of circ-FANCA in LPS-induced HK2 cells transfected with circ-FANCA or pCD5-ciR was determined by qRT-PCR assay. **i** MiR-93-5p level was determined through qRT-PCR in LPS-induced HK2 cells following the transfection of si-NC, si-circ-FANCA, pCD5-ciR or circ-FANCA. **P < 0.01, ***P < 0.001, ****P < 0.0001

To probe the underlying mechanism of circ-FANCA in the regulation of LPS-induced HK2 cell injury, online software starBase v2.0 was used to identify the potential target miRNAs of circ-FANCA. As depicted in Fig. 3a, circ-FANCA contained a putative complementary sequence of miR-93-5p, indicating that miR-93-5p might be a target of circ-FANCA. To validate this prediction, the dual-luciferase reporter assay and RIP assay were conducted. First, the overexpression efficiency of miR-93-5p was verified in HK2 cells (Fig. 3b). The dual-luciferase reporter assay revealed that miR-93-5p transfection distinctly inhibited the luciferase activity of WT-circ-FANCA in HK2 cells, but did not affect the luciferase activity of MUT-circ-FANCA (Fig. 3c). Meanwhile, RIP assay showed that miR-93-5p and circ-FANCA were all enriched in Ago2 protein complexes in HK2 cells compared to IgG control group, further confirming the combination between miR-93-5p and circ-FANCA (Fig. 3d). Thereafter, the expression of miR-93-5p in septic AKI patients and LPS-induced HK2 was determined by qRT-PCR assay. The results showed that miR-93-5p level was markedly declined in the serums of septic AKI patients compared to corresponding healthy patients (Fig. 3e). Additionally, Spearman’s correlation coefficient analysis showed that the levels of miR-93-5p and circ-FANCA were negatively correlated in the serums of septic AKI patients (Fig. 3f). Moreover, miR-93-5p expression was impeded by LPS treatment in a concentration-dependent form in HK2 cells (Fig. 3g). To assure the relationship between miR-93-5p and circ-FANCA, the overexpression vector of circ-FANCA was transfected into HK2 cells. As expected, LPS treatment elevated circ-FANCA level in HK2 cells, and the transfection of circ-FANCA further accelerated the effect, indicating the success transfection of circ-FANCA (Fig. 3h). Moreover, circ-FANCA knockdown dramatically elevated miR-93-5p expression, while circ-FANCA overexpression apparently decreased miR-93-5p expression in LPS-treated HK2 cells (Fig. 3i). These results demonstrated that circ-FANCA negatively modulated miR-93-5p expression by directly targeting.

### MiR-93-5p inhibition restored the effects of circ-FANCA knockdown on cell damage inhibition in LPS-stimulated HK2 cells

**Fig. 4:**
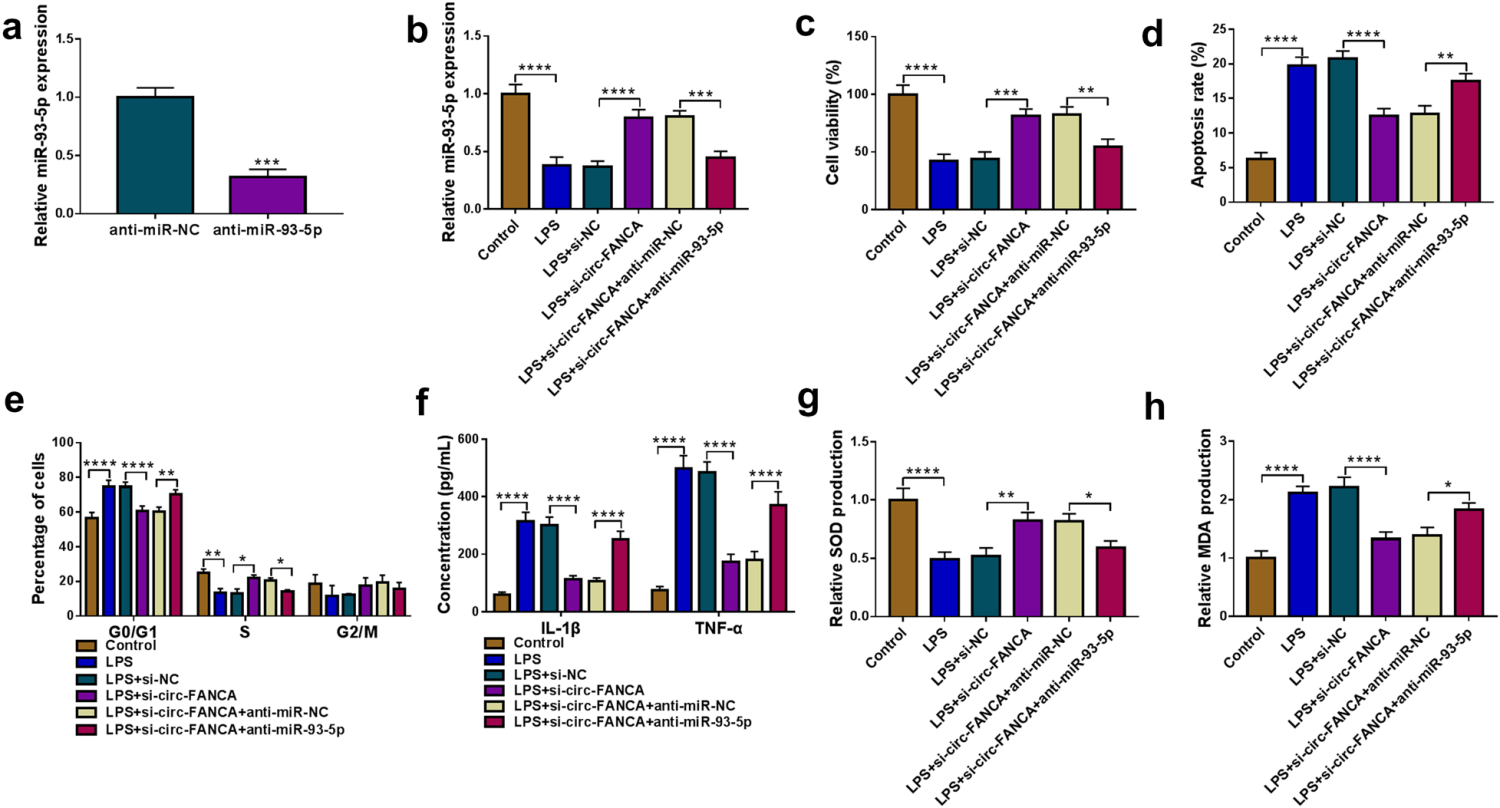
Circ-FANCA regulated LPS-induced HK2 cell injury by targeting miR-93-5p. **a** The knockdown efficiency of anti-miR-93-5p was analyzed by qRT-PCR. HK2 cells were divided into 6 groups: Control, LPS, LPS+ si-NC, LPS+ si-circ-FANCA, LPS+ si-circ-FANCA +anti-miR-NC and LPS+ si-circ-FANCA+ anti-miR-93-5p. **b** The qRT-PCR was performed for miR-93-5p expression detection in the above groups. **c**–**e** The measurement of cell viability (**c**), cell apoptosis (**d**), cell cycle distribution (**e**) was carried out via CCK-8 assay or flow cytometry in the above groups. **f** The release of IL-1β and TNF-α was investigated by ELISA. **g**, **h** The activities of SOD and MDA in these cells were determined using the corresponding kits. *P < 0.05, **P < 0.01, ***P < 0.001, ****P < 0.0001

To determine whether miR-93-5p was a molecular mediator of circ-FANCA in LPS-induced HK2 cell injury, rescue experiments were performed to monitor whether miR-93-5p inhibition could rescue the effects of circ-FANCA knockdown. LPS-treated HK2 cells were transfected with si-NC, si-circ-FANCA, si-circ-FANCA + anti-miR-NC or si-circ-FANCA + anti-miR-93-5p. As exhibited in Fig. 4a, miR-93-5p expression was visibly depressed in HK2 cells transfected with anti-miR-93-5p, hinting the high knockdown efficiency of anti-miR-93-5p. Meanwhile, si-circ-FANCA-triggered miR-93-5p increase was evidently reversed by anti-miR-93-5p transfection in LPS-treated HK2 cells (Fig. 4b). Functionally, circ-FANCA knockdown restrained LPS-suppressed cell viability and cycle progress, which were restored by the reintroduction of anti-miR-93-5p (Fig. 4c and e). Concurrently, LPS-induced increase of apoptotic rate was largely weakened by si-circ-FANCA addition in LPS-treated HK2 cells, while miR-93-5p inhibition elevated the apoptotic rate (Fig. 4d). ELISA data indicated that si-circ-FANCA-induced reduction of IL-1β and TNF-α levels in LPS-induced HK2 cells was attenuated by miR-93-5p silence (Fig. 4f). Besides, miR-93-5p inhibition attenuated the effects of circ-FANCA silence on the levels of SOD and MDA in LPS-activated HK2 cells (Fig. 4g and h). Collectively, all data illustrated that circ-FANCA interference alleviated LPS-mediated HK2 cell damage by binding to miR-93-5p.

### OXSR1 was identified as a direct target gene of miR-93-5p

**Fig. 5:**
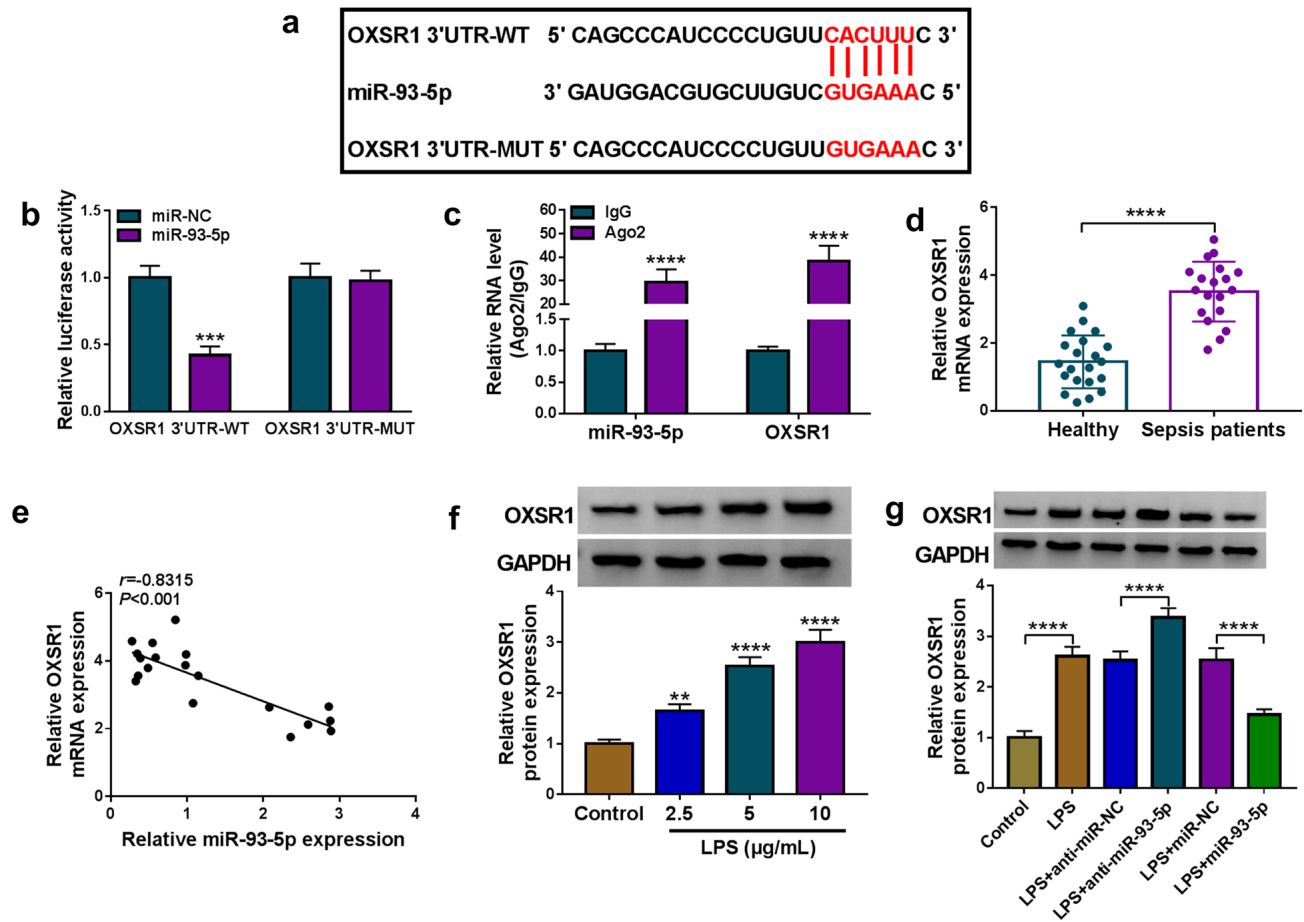
OXSR1 was a direct target of miR-93-5p. **a** The potential binding sites between miR-93-5p and OXSR1 were exhibited. **b**, **c** Dual-luciferase reporter assay and RIP assay were performed to verify whether OXSR1 could interact with miR-93-5p. **d** The mRNA level of OXSR1 in the serums of septic AKI patients (n = 19) and healthy controls (n = 19) was measured by qRT-PCR assay. **e** The correlation between OXSR1 mRNA and miR-93-5p in the serums of septic AKI patients was analyzed by Spearman’s correlation coefficient analysis. **f** The protein expression of OXSR1 in HK2 cells treated with different concentrations of LPS was quantified by western blot. **g** The protein level of OXSR1 in LPS-induced HK2 cells with miR-93-5p knockdown or overexpression was tested by western blot. **P < 0.01, ***P < 0.001, ****P < 0.0001

Based on online software starBase v2.0, OXSR1 was revealed to be a molecular target of miR-93-5p and their potential binding region was shown in Fig. 5a. The dual-luciferase reporter assay displayed that the luciferase activity of OXSR1 3′UTR-WT in HK2 cells was visibly inhibited after transfection of miR-93-5p, while the luciferase activity of OXSR1 3′UTR-MUT was insusceptible by miR-93-5p introduction (Fig. 5b). Meanwhile, the elevated levels of miR-93-5p and OXSR1 in Ago2 protein complexes of RIP assay in HK2 cells further demonstrated the interaction between miR-93-5p and OXSR1 (Fig. 5c). Furthermore, OXSR1 level was enhanced in the serums of septic AKI patients compared to healthy controls (Fig. 5d). And there was an inverse correlation between the expression of miR-93-5p and OXSR1 in the serums of patients with sepsis (Fig. 5e). Moreover, OXSR1 protein level was strongly elevated in LPS-treated HK2 cells compared to control cells (Fig. 5f). In addition, miR-93-5p inhibition remarkably increased the protein level of OXSR1 in LPS-treated HK2 cells, while miR-93-5p overexpression exhibited the opposite results (Fig. 5g). Thus, it was concluded that miR-93-5p negatively regulated OXSR1 expression by direct interaction.

### Overexpression of miR-93-5p facilitated cell viability and repressed cell apoptosis, inflammation and oxidative stress in LPS-treated HK2 cells by targeting OXSR1

**Fig. 6:**
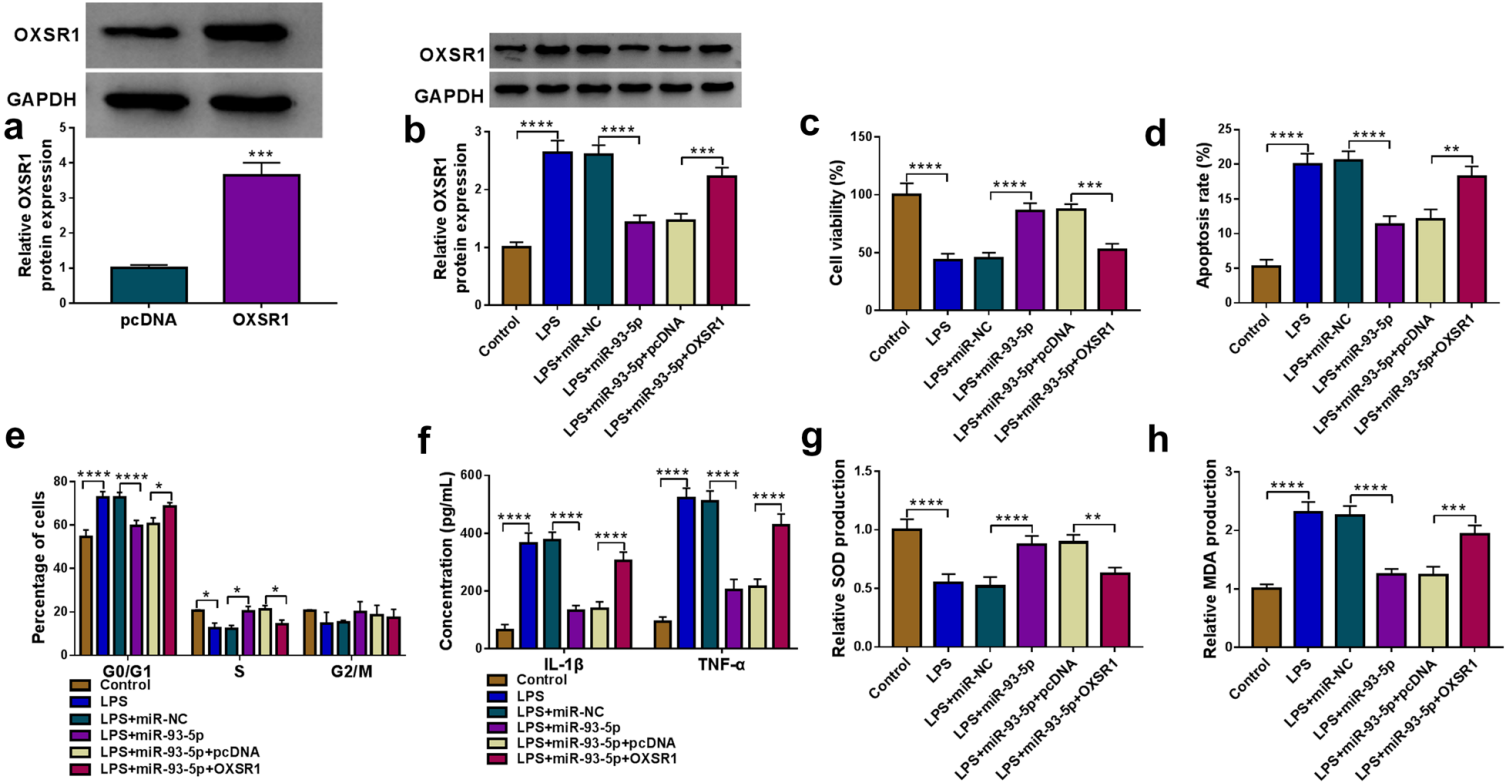
Overexpression of miR-93-5p alleviated LPS-induced cell injury in HK2 cells by targeting OXSR1. **a** The protein level of OXSR1 in HK2 cells transfected with pcDNA or OXSR1 was measured by western blot. **b**–**h** LPS-treated HK2 cells were transfected with miR-NC, miR-93-5p, miR-93-5p+pcDNA or miR-93-5p+OXSR1. **b** The protein level of OXSR1 in LPS-induced HK2 cells after transfection was tested by western blot. **c** Cell viability in these cells was assessed by CCK-8 assay. **d**, **e** Cell apoptosis and percentage of cells at different stages were monitored by flow cytometry assay. **f** The concentration of IL-1β and TNF-α was examined by ELISA using corresponding kits. **g**, **h** The levels of SOD and MDA in these cells were examined by corresponding specific kits. *P < 0.05, **P < 0.01, ***P < 0.001, ****P < 0.0001

For further elucidating the connection between miR-93-5p and OXSR1 in LPS-induced HK2 cell injury, HK2 cells were transfected with miR-93-5p, miR-93-5p + OXSR1 or corresponding controls. As observed in Fig. 6a, the transfection of OXSR1 overexpression vector resulted in a distinct elevation in OXSR1 protein level in HK2 cells. Western blot assay showed that miR-93-5p transfection significantly reduced the protein level of OXSR1 in LPS-treated HK2 cells, while the introduction of OXSR1 overexpression vector overturned the impact (Fig. 6b). In addition, the miR-93-5p overexpression-mediated pro-proliferation (Fig. 6c and e), anti-apoptosis (Fig. 6d), anti-inflammation (Fig. 6f) and anti-oxidation (Fig. 6g and h) impacts were evidently ameliorated by elevating OXSR1 expression in LPS-injured HK2 cells. To sum up, these outcomes suggested that miR-93-5p overexpression attenuated the LPS-induced HK2 cell injury by sponging OXSR1.

### Circ-FANCA positively regulated OXSR1 expression by sponging miR-93-5p

**Fig. 7:**
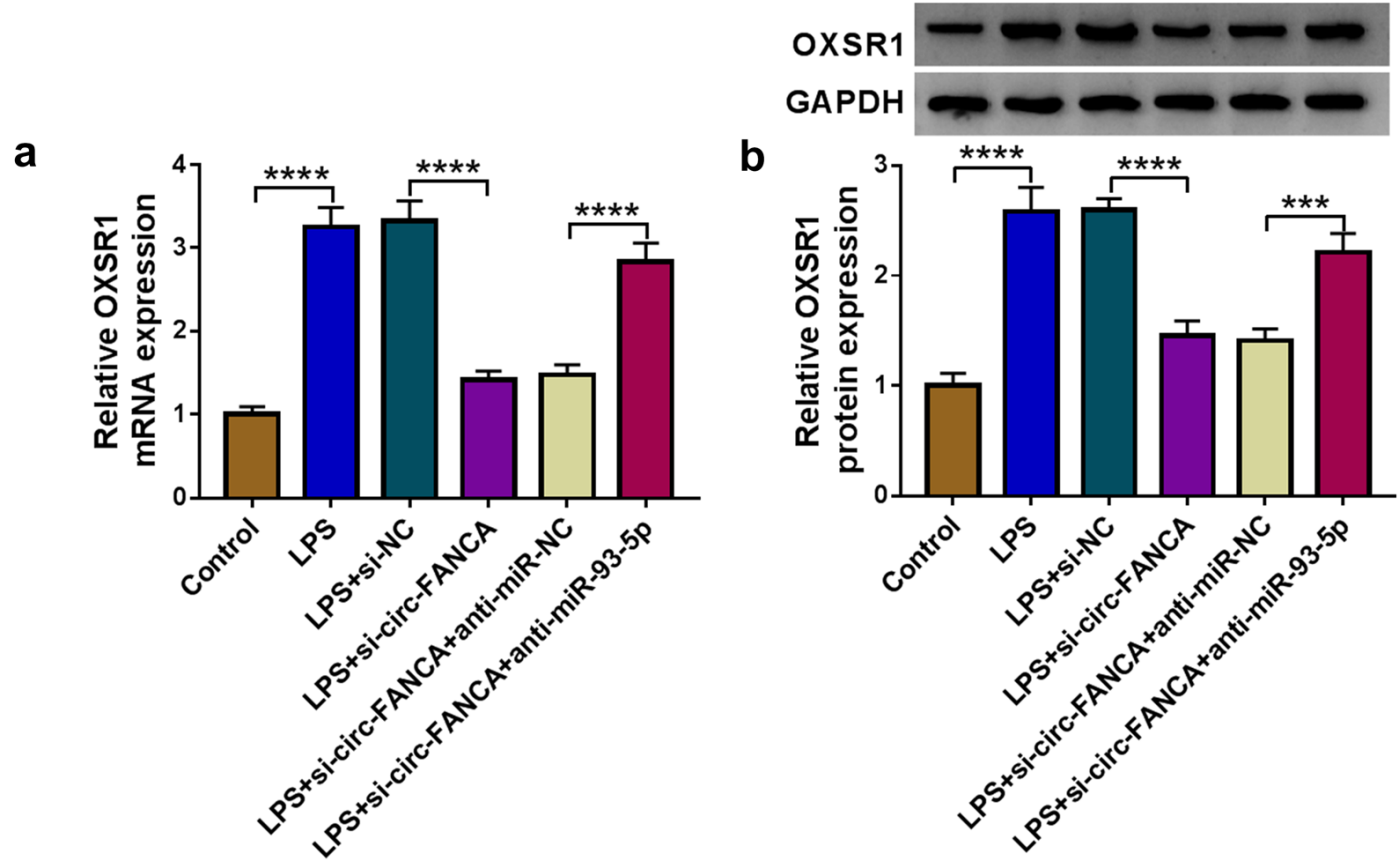
Circ-FANCA positively modulated OXSR1 expression by targeting miR-93-5p. **a**, **b** After transfected with si-circ-FANCA, si-circ-FANCA+anti-miR-93-5p or corresponding controls, the mRNA and protein levels of OXSR1 in LPS-induced HK2 cells were measured by qRT-PCR and western blot assays. ***P < 0.001, ****P < 0.0001

Finally, the associations among circ-FANCA, miR-93-5p and OXSR1 were further investigated. The mRNA and protein expression of OXSR1 was remarkably lessened in LPS-induced HK2 cells transfected with si-circ-FANCA, while the effect was reversed in cells transfected with si-circ-FANCA + anti-miR-93-5p (Fig. 7a and b). All the evidence indicated that circ-FANCA sponged miR-93-5p to positively regulate the expression of OXSR1.

### Exosomal circ-FANCA was upregulated in LPS-induced HK2 cells

**Fig. 8:**
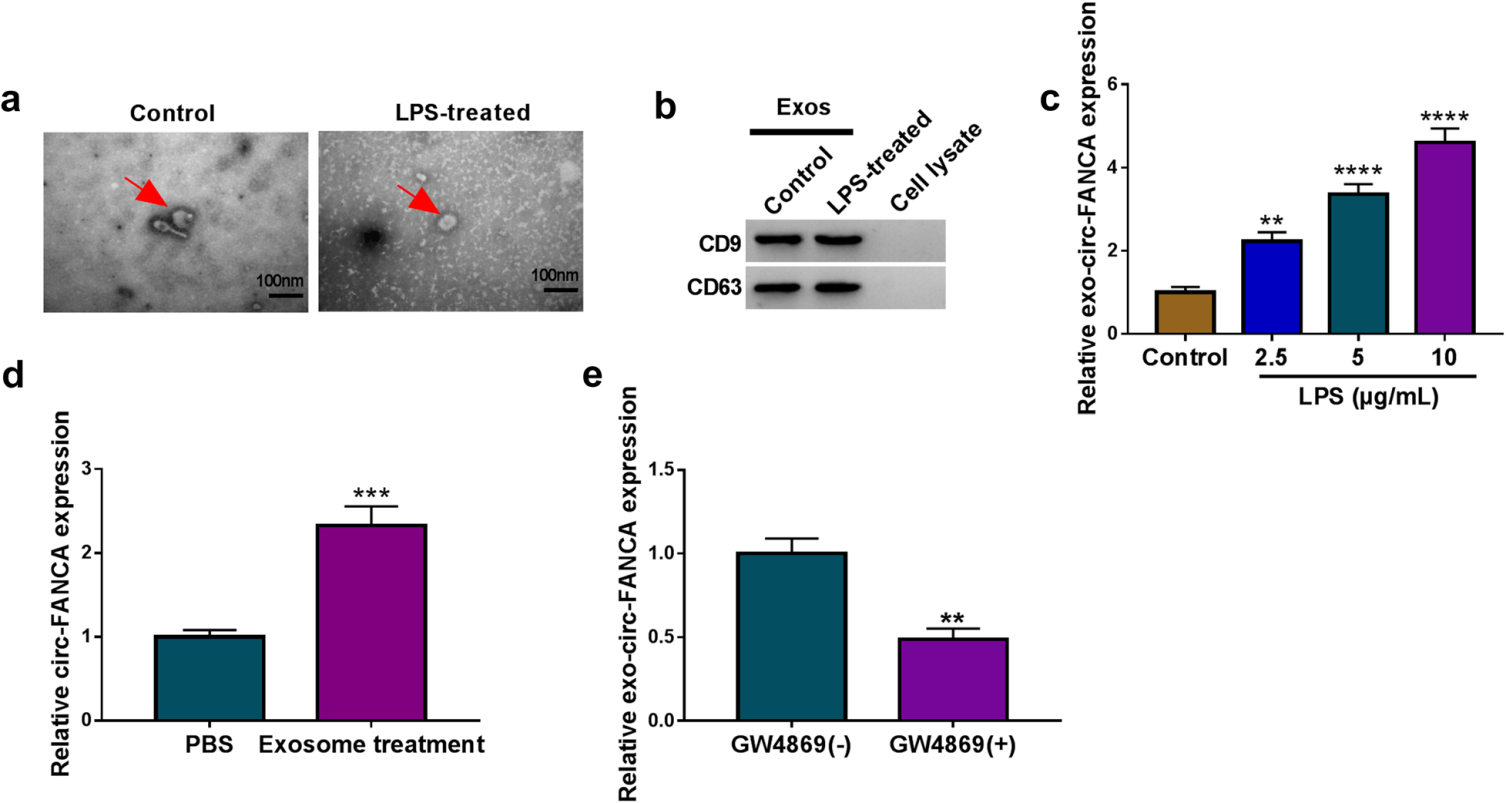
Exosomes derived from LPS-induced HK2 cells mediated the release of circ-FANCA. **a** Electron micrograph of exosomes isolated from the culture medium of LPS-treated HK2 cells or untreated HK2 cells. The scale bar represents 100 nm. **b** Western blot analysis for exosome markers (CD9 and CD63) in HK2 cells. **c** QRT-PCR assay for the expression level of exo-circ-FANCA in HK2 cells treated with LPS. **d** QRT-PCR assay for the expression of circ-FANCA in HK2 cells co-incubated with exosomes derived from LPS-induced HK2 cells or PBS. **e** QRT-PCR assay for the expression of exo-circ-FANCA in LPS-treated HK2 cells co-incubated with GW4869 or not. **P < 0.01, ***P < 0.001, ****P < 0.0001

To identify the function of exosomes in the progression of LPS-induced cell damage, the exosomes were isolated from LPS-treated HK2 cells. The representative images of isolated exosomes were displayed in Fig. 8a, the exosomes isolated from LPS-treated HK2 cells possessed the similar rounded characteristics. Next, western blot analysis declared that the protein levels of exosomal markers CD9 and CD63 were detectable in exosomes from LPS-disposed HK2 cells (Fig. 8b), suggesting the successful isolation of exosomes. Meanwhile, qRT-PCR assay confirmed that the level of exosomal circ-FANCA (exo-circ-FANCA) derived from HK2 cells exposed to LPS was signally upregulated in a dose-dependent manner (Fig. 8c). Then, it was investigated whether circ-FANCA could be delivered through exosomes derived from LPS-treated HK2 cells. HK2 cells were disposed with PBS or exosomes. The result suggested that exosome treatment evidently enhance circ-FANCA expression in untreated HK2 cells (Fig. 8d). Additionally, GW4869 was added into LPS-induced HK2 cells to evaluate the effects of exosomal inhibition on circ-FANCA expression. The result revealed that the level of exo-circ-FANCA was remarkably inhibited by GW4869 treatment in LPS-induced HK2 cells (Fig. 8e). Taken together, these data implied that exosomes derived from LPS-treated HK2 cells were involved in the delivery of circ-FANCA in vitro.

## Discussion

As a global healthcare concern, sepsis-induced AKI may evoke shock and death to a certain degree [23]. Although growing research into this disease, the pathogenesis is not fully elaborated. Therefore, numerous studies constructed LPS-induced AKI cell models and claimed the associated events such as inflammatory response and oxidative stress in AKI development [24, 25]. The present report established the renal injury models by treating HK2 cells with LPS to explore the role of circ-FANCA and the underlying mechanism in sepsis-associated AKI in vitro.

Increasing studies have demonstrated that many newly identified circRNAs are implicated in the regulation of sepsis-induced AKI [26]. Shi et al. discovered that circPRKCI reduced LPS-induced HK2 apoptosis and inflammatory injury through the upregulation of miR-545-mediated ZEB2 [27]. CircANRIL silence was reported to attenuate LPS-induced apoptosis, inflammatory responses and oxidative stress in HK2 cells by targeting the miR-9 [28]. Additionally, circ-FANCA was documented to be highly expressed in sepsis-induced AKI [18]. Even so, the exact impacts of circ-FANCA need to be further explored. In this study, we monitored that circ-FANCA was an upregulated circRNA in serum specimens from septic AKI patients and LPS-activated HK2 cells. In the biological function, circ-FANCA downregulation reversed the LPS-inhibited cell viability, cell cycle progress and LPS-promoted cell apoptosis, inflammatory response and oxidative stress in LPS-treated HK2 cells. Thus, these observations implied that circ-FANCA served as a promoting factor in the progression of sepsis-induced AKI, and circ-FANCA silence was able to alleviate LPS-induced injury in HK2 cells.

The regulatory function of circRNA/miRNA/mRNA axis in multifarious diseases has been affirmed [29, 30]. Meanwhile, circRNAs have been certified to regulate sepsis progression via sponging miRNAs [26]. However, the studies about the circRNA/miRNA pathway in sepsis-induced AKI were insufficient. MiR-93-5p was reported to be downregulated in LPS-treated HK2 cells, and participated in sepsis-induced AKI via KDM6B/H3K27me3/TNF-α axis [22]. Here, after bioinformatical prediction and the binding validation using dual-luciferase reporter assay and RIP assay, miR-93-5p was considered as a miRNA target of circ-FANCA, and it was low expressed in septic AKI patients and LPS-injured HK2 cells. Meanwhile, circ-FANCA had negative regulatory effect on miR-93-5p expression, proving that miR-93-5p was a downstream target for circ-FANCA. To explore the regulatory mechanism of circ-FANCA/miR-93-5p pathway, rescue experiments in LPS-treated HK2 cells were conducted in our study. And the results presented that miR-93-5p silence could rescue the inhibitory effects on HK2 cell damage caused by circ-FANCA knockdown. Thus, we speculated that circ-FANCA might be implicated in LPS-induced AKI via sponging miR-93-5p.

CircRNAs have been revealed to compete with mRNAs for miRNAs binding in the cytoplasm thus to interfere downstream gene expression [31]. Nevertheless, it is unknown whether OXSR1 was involved in the circRNA/miRNA regulatory network of circ-FANCA. In the present study, OXSR1 was confirmed to be a potential target of miR-93-5p. As one serine/threonine kinase family member, OXSR1 could regulate downstream kinases responding to environmental stress and is accountable to ion co-transportation in the kidney [32–34]. OXSR1 was unraveled to be abnormally upregulated in hepatocellular carcinoma (HCC) and its increase predicted poor prognosis and promoted HCC tumorigenesis [35]. Nevertheless, the research about the function of OXSR1 in septic AKI is lacking. It has been manifested that OXSR1 level was elevated in septic AKI rat models and OXSR1 played a promoting role in the progression of septic AKI [36]. Consistent with this finding, in this paper, OXSR1 was identified to be notably upregulated in serum specimens from septic AKI patients and LPS-challenged HK2 cells. In function, OXSR1 introduction abrogated miR-93-5p overexpression-blocked LPS-induced HK2 cell injury. Furthermore, circ-FANCA regulated OXSR1 expression by sponging miR-93-5p. Therefore, circ-FANCA functioned as a regulatory molecule in sepsis-engendered AKI via downregulating miR-93-5p and upregulating OXSR1.

Exosomes, biological lipid bilayer nanoparticles secreted via the endosomal pathway of cells, have recently emerged as important cargos that carry multiple mediators critical for the pathogenesis of sepsis-associated organ dysfunctions [37]. Meanwhile, the application of exosomes in sepsis may be a novel approach for sepsis treatment [37–39]. Recently, circular RNAs have been identified for their enrichment and stability in exosomes, and exosomal circRNAs have potential applications as biomarkers and novel therapeutic targets in human diseases [40]. Herein, exosomal circ-FANCA level was raised in LPS-induced HK2 cells in a dose-dependent manner. Meanwhile, circ-FANCA could be delivered through exosomes in LPS-treated HK2 cells, and GW4869 addition hindered the expression of exosomal circ-FANCA. Therefore, we speculated that targeting circ-FANCA may be effective therapeutic method for sepsis-induced AKI remedy.

## Conclusion

Collectively, circ-FANCA and OXSR1 expression levels were elevated in serum specimens of septic AKI patients and LPS-induced septic AKI cell models. Circ-FANCA knockdown attenuated LPS-induced HK2 cell injury, including proliferation, apoptosis, inflammatory responses and oxidative stress, by modulating the miR-93-5p/OXSR1 signaling pathway. The findings could help us to understand the pathogenic mechanism of septic AKI and might offer a novel feasible therapeutic target to improve the treatment of AKI incurred by sepsis.

## Data Availability

The data sets used and/or analyzed during the current study are available from the corresponding author on reasonable request.
